# THE ROLE OF SECURED AND UNSECURED DEBT IN RETIREMENT PLANNING AMONG PRE-RETIREES

**DOI:** 10.1093/geroni/igz038.1404

**Published:** 2019-11-08

**Authors:** Zibei Chen, Karen Zurlo

**Affiliations:** 1 University of Michigan, Ann Arbor, Michigan, United States; 2 Rutgers, New Brunswick, New Jersey, United States

## Abstract

The retirement landscape is transformed by the shifting of risk and responsibility to individuals, who are increasingly responsible for their retirement security. Many factors lead to indebted and overleveraged American households. Specifically, nearly 40% of Americans approaching retirement are heavily indebted. Understanding the role of secured and unsecured debt in retirement planning becomes an urgent concern because debt is highly related to well-being in retirement among a growing number of older Americans. We focus on pre-retirees because these individuals have time to earn an income and plan ahead before they commit to a fully retired lifestyle. Utilizing data from the 2015 National Financial Capability Study, we identified the secured and unsecured debt that influences retirement planning among a national sample of pre-retirees, aged 51 to 61 years. Regression and mediation analyses were used to examine the relationship between debt and retirement planning and to identify the mediating effect of having a retirement account on the relationship between unsecured and secured debt and retirement planning. Our results indicated that mortgage debt and credit card debt were negatively associated with retirement planning. Having a retirement account is positively associated with retirement planning and it also mediates the relationship between credit card debt, specifically, and retirement planning. In conclusion, we urge individuals and financial planning executives to take time during the pre-retirement years to assess various forms of debt and determine how it is affecting their retirement planning objectives. And policy-makers should address the challenges faced by indebted pre-retirees.

